# Empirical Derivation and Prediction of Treatment Trajectories in Harmonized AUD Clinical Trial Datasets

**DOI:** 10.1111/adb.70069

**Published:** 2025-07-23

**Authors:** Robert J. Kohler, Yasmin Zakiniaeiz, Terril L. Verplaetse, C. Leonardo Jimenez Chavez, MacKenzie R. Peltier, Hang Zhou, Sherry A. McKee, Walter Roberts

**Affiliations:** ^1^ Department of Psychiatry Yale University School of Medicine New Haven Connecticut USA; ^2^ Mental Health Service Line VA Connecticut Healthcare System West Haven Connecticut USA; ^3^ Division of Clinical Neurosciences National Center for PTSD New Haven Connecticut USA

**Keywords:** alcohol use disorder, machine learning, randomized controlled trial, treatment

## Abstract

In clinical settings targeting alcohol use disorder (AUD), it is often unclear whether a treatment option may best suit a patient's clinical needs. Clinicians providing AUD treatment are often required to predict patients' responses to guide treatment decisions. Recently, machine learning approaches have been used as tools in precision medicine to help guide these clinical decisions. However, the extent of their clinical utility in populations undergoing treatment is largely unknown. Using data from four Phase 2 randomized clinical trials affiliated with the NIAAA Clinical Investigations Group and a Phase 3 trial sponsored by the NIAAA, we developed a machine learning model to predict treatment response phenotypes derived from clustering drinking rates at the end of treatment. Harmonized data included demographics and baseline data from biological and clinical assessments. Follow‐up analyses were performed to characterize treatment response phenotypes. Three clusters corresponding to mild (M_SDU_ = 1.3), moderate (M_SDU_ = 6.70) and severe (M_SDU_ = 15.3) alcohol consumption were identified from end‐of‐treatment drinking data. Performance of the tree‐based classifier using out‐of‐sample test data was 71% with baseline drinking included and 61% without. Exploratory analyses revealed participants clustered as mild drinkers showed reductions in drinking across treatment (*M*_*Difference*_ = −0.731, SE = 0.114, *p* < 0.001) whereas participants clustered as severe had escalation in use (*M*_*Difference*_ = 6.82, SE = 0.52, *p* < 0.001). Although males drank more than females at baseline (*M*_*Difference*_ = 1.46, SE = 0.287, *p* < 0.001), no significant differences in consumption emerged at the end of treatment. Findings from this work indicate that alcohol use derived from patterns of consumption at the end of treatment maps onto unique treatment response trajectories for mild and severe forms of AUD. Furthermore, the identified clusters revealed sex‐specific differences in alcohol consumption patterns across different phases of treatment. Overall, this highlights the utility of computational methods for deriving clinically meaningful AUD‐related phenotypes across multiple studies, each with different treatments and participant characteristics.

## Graphical abstract

Using a data‐driven approach, we identified three clusters of alcohol consumption at the end of treatment for AUD that were associated with unique drinking trajectories and sex‐specific differences. We predicted these clusters using clinical feature data and highlight the utility of data‐driven methods for deriving AUD‐related phenotypes.

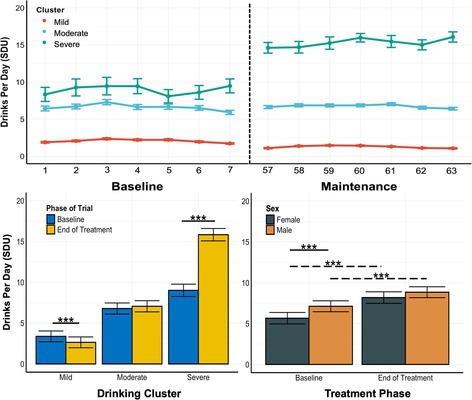

## 1 Introduction

Alcohol use disorder (AUD) remains a significant public health challenge, characterized by diverse patterns of alcohol use and varying responses to treatment [1, 2]. Traditional approaches to classifying treatment response in AUD have predominantly relied on broad, categorical outcomes, often failing to capture the nuanced trajectories of individual patients' changes during treatment [1, 3]. Effectively identifying these more nuanced treatment trajectories may facilitate outcome tracking during clinical trials to better link a particular pattern of consumption with a unique AUD phenotype and to facilitate regulatory decision making [4, 5]. Currently, treatment response is most often framed in the context of Food and Drug Administration (FDA) identified clinical trial outcomes, which define treatment success as the absence of any alcohol use (i.e., percent participants abstinent) or the absence of heavy drinking (i.e., percent participants with no heavy drinking days) [6, 7]. While these endpoints are useful in that they provide a standardized framework for evaluating the efficacy of treatments, they frequently fail to capture clinically meaningful differences in how people respond to treatment [8]. This limitation is in part why the FDA has recently accepted the World Health Organization's (WHO) risk level definitions for alcohol consumption. These levels (i.e., low, medium, high and very high) provide stratification of alcohol risk beyond just abstinence or heavy drinking [9]. Indeed, prior work has shown that one and two‐level reductions in these risk levels are associated with clinically meaningful outcomes [3]. Together, this underscores the need for innovative approaches to more accurately represent the complexities of response trajectories during AUD treatment.

The application of novel, computationally driven statistical methods may improve our understanding of treatment responses, capturing nuanced patterns of change over time that are often overlooked by traditional AUD treatment outcome measures [10, 11]. Such techniques leverage individual variation in trajectories of alcohol use and can account for patterns of use that are nonlinear or highly heterogeneous across individuals [8]. Thus, these approaches may offer a more detailed map of the recovery process, highlighting the variable nature of treatment progress. An inherent barrier to the application of such statistical modeling approaches to characterize treatment response is that these techniques are ‘data hungry’, requiring thousands of observations to achieve more realistic and generalizable effect size estimates [12, 13]. With few notable exceptions, clinical trials of novel treatments for AUD typically do not exceed 1 to 200 participants [14]. To attempt to overcome this limitation, we have undertaken a harmonization of datasets across the National Institute on Alcohol Abuse and Alcoholism's Clinical Investigation Group (NCIG), pooling data from various clinical trials [15, 16, 17, 18, 19] to amass a large, diverse sample that is necessary for identifying and predicting patterns of alcohol use during treatment. By utilizing a harmonized dataset aggregated from multiple AUD clinical trials, this study provides a robust platform for analysis, enhancing the generalizability and applicability of the findings.

This effort may aid in enhancing the granularity with which we can assess and interpret the subtleties of treatment responses, thereby producing more reliable and reproducible findings. In this context, the current study utilizes a cross‐validated, machine learning approach, with out‐of‐sample generalizability testing, to predict AUD treatment response patterns that are derived from a timeseries‐based clustering algorithm. This approach not only takes advantage of underlying patterns of drinking to identify distinct groups of treatment responders but also to identify baseline predictors of these trajectories. From this, we aim to shed light on the complex landscape of AUD treatment response, providing a novel lens through which the effectiveness of interventions can be better evaluated and optimized. This methodology holds potential not only for advancing the theoretical understanding of AUD recovery but also for informing personalized treatment strategies, ultimately contributing to improved patient outcomes. To this end, we also use a cutting‐edge machine learning framework to identify clinical features prior to treatment onset that may predict future treatment trajectories. Prior studies using machine learning approaches have successfully predicted treatment‐related outcomes for each of these trials individually [5, 20, 21, 22], but that have not yet been combined into a more heterogeneous sample.

Based on previous findings [5, 8], we hypothesize that clustering individuals based on their rates of drinking at the end of treatment will yield distinct trajectory groups that map to drinking severity at the end of treatment. We further hypothesize that these identified clusters will be modestly predicted by clinical and biological data that was collected at baseline of the clinical trials [21]. In particular, these primary drivers of model performance will map to baseline rates of drinking [5, 21, 22], drinking consequences (i.e., a proxy for AUD severity) and liver functioning. Given well‐documented differences in the expression of AUD among men and women [23], we also hypothesize that participant sex will be an additional driver of model performance. Findings from this work have the potential to pave the way for more targeted and effective treatment modalities, aligning with the growing emphasis on personalized medicine in the field of addiction.

## 2 Methods

### 2.1 Data Source

Data were obtained through the National Institute on Alcohol Abuse and Alcoholism's Clinical Investigations Group (NCIG) data‐sharing programme. Participant‐level data from five multisite, randomized controlled trials (RCT) designed to evaluate the efficacy of pharmacological interventions for the treatment of alcohol dependence and AUD (NCT01613014, NCT00970814, NCT01146613, NCT00006206 and CSP1027) comprised the data sources. In all five RCTs, treatment was delivered on an outpatient basis for approximately 3 months, and standardized design elements and outcome assessment procedures were utilized. All five RCTs also included a placebo group, and all participants received medical management or brief behavioural intervention in addition to the medication—or placebo—treatment. The following medications were assessed for their efficacy in reducing AUD severity across the trials, including naltrexone (NCT00006206, *n* = 154), acamprosate (NCT00006206, *n* = 152), naltrexone + acamprosate (NCT00006206, *n* = 148), quetiapine (CSP1027, *n* = 104), gabapentin (NCT01613014, *n* = 165), levetiracetam (NCT00970814, *n* = 50) and varenicline (NCT01146613, *n* = 86). NCT00006206 also included forms of combined behavioural therapy (CBI) alone (*n* = 157) and a subset of separate individuals receiving naltrexone (*n* = 155), acamprosate (*n* = 151) and the combination (*n* = 157). Additional details for each study can be found in Table S1. Data from 1931 participants were available after removing incomplete data (*n* = 144 removed). Table S1 displays sample characteristics for each RCT separately.

### 2.2 Data Harmonization and Feature Inclusion

For this purpose‐driven investigation, we identified common data elements that were included across clinical trials and included only these elements in the aggregated dataset. Because of the overlap in clinical trial methodologies, many of the assessment instruments were identical across trials, including both self‐report (e.g., drinking consequences) and laboratory‐based (e.g., liver enzyme biomarkers) assessments. However, inconsistencies between trials did exist in the survey methods for several assessments, and therefore, many assessments could not be included in the harmonized dataset. Due to these inconsistencies, a composite score was created for an index of depression and anxiety. To accomplish this, we identified separate assessments in each trial that pertained to aspects of depression and anxiety, summed the scores of these items and scaled the new composite score for each RCT separately before collating the data across the RCTs. This composite score included items from the Short Form‐12 assessment (NCT01146613), the Hamilton Anxiety Scale and Montgomery Asberg Depression Rating Scale (NCT00970814; CSP1027), the Beck Depression and Anxiety Indices (NCT01613014) and the Brief Symptom Inventory (NCT00006206). Several categorical variables were harmonized by collapsing levels of certain variables (e.g., combining income brackets, marital status; see Supporting information material for details). The RCTs also varied in terms of treatment length and availability of self‐reported drinking outcomes during the trials. To preserve the harmonized structure of the dataset, our primary outcome (e.g., drinking clusters) was derived from drinking data that was limited to the last week of treatment maintenance for the clinical trial with the fewest number of treatment days, corresponding to Days 57 through 63 for each trial.

A total of 19 baseline assessments (i.e., physiological and clinical) and demographic characteristics were included as features in our analysis. We refer to baseline assessments as the first time an assessment was administered following the start of an RCT and any required abstinence. Clinical assessments included a depression and anxiety composite, assessments of withdrawal derived from the Clinical Institute Withdrawal Assessment for Alcohol (CIWA) [24] and drinking consequences from the Drinker Inventory of Consequences (DrinC) [25] survey. Physiological and laboratory‐based assessments included blood pressure (e.g., systolic and diastolic), pulse rate, weight and blood tests for creatine, gamma‐glutamyl transferase (GGT), alanine transaminase (ALT), aspartate aminotransferase levels (AST) and total bilirubin. Demographic information, including age, sex, years of education, individual annual income, marital status, treatment received and drinking rates during the first week of each trial, was also used as features in the machine learning model.

### 2.3 Identification of Drinking Patterns During Treatment

To identify baseline predictors of treatment response, we first combined self‐reported drinking data from Days 57 to 63 of each study's maintenance phase and removed participants who did not have complete drinking data (*n*_Remaining_ = 2072). This treatment period was selected as it corresponded to the final week of treatment maintenance for one of the included trials, thus allowing us to identify clusters across all studies that were based on a similar number of total treatment days. Next, we used dynamic time warping (DTW) [26, 27], a timeseries‐based approach, to cluster individuals based on the number of standard drinking units (SDUs) consumed on each of the 7 days of the treatment maintenance phase. Consistent with previous findings [10, 11], we a priori selected a three‐cluster solution for the DTW and identified the degree of overlap using the context‐independent optimality and partiality (COP) distance index [28]. This was compared to the COP index of a two‐cluster solution to determine the adequacy of our a priori chosen solution. Table 1 displays combined sample characteristics across the identified clusters for demographics, and Table 2 displays characteristics for the clinical, biological and physiological assessments that were included.

**TABLE 1:** Participant demographic characteristics.

		Cluster		
		Mild (*n* = 1531)	Moderate (*n* = 299)	Severe (*n* = 101)
Age		45.3 (10.6)	47.91 (10.9)	45.7 (9.9)
Education (years)		14.5 (2.7)	14.9 (2.8)	13.7 (2.4)
Baseline drinks (7 days)		2.0 (3.3)	6.5 (4.3)	8.9 (8.9)
Sex	Female	476 (31%)	86 (29%)	19 (19%)
	Male	1055 (69%)	213 (71%)	82 (81%)
Employment	Unemployed/other	558 (36%)	84 (28%)	42 (42%)
	Working	973 (64%)	215 (72%)	59 (58%)
Marriage status	Married	653 (43%)	146 (49%)	25 (25%)
	Not married	878 (57%)	153 (51%)	76 (75%)
Annual income	$30 001–$60 000	414 (27%)	65 (22%)	25 (25%)
	< $30 000	418 (27%)	70 (23%)	47 (47%)
	> $60 000	676 (44%)	158 (53%)	28 (28%)
	Not provided	23 (2%)	6 (2%)	1 (< 1%)
Race	Non‐White	406 (27%)	48 (16%)	32 (32%)
	White	1125 (73%)	251 (84%)	69 (68%)
Ethnicity	Hispanic	134 (9%)	20 (7%)	14 (14%)
	Non‐Hispanic	1397 (91%)	279 (93%)	87 (86%)
Randomized controlled trial	COMBINE (NCT00006206)	1071 (70%)	87 (29%)	58 (57%)
	Gabapentin (NCT01613014)	187 (12%)	98 (33%)	10 (10%)
	Levetiracetam (NCT00970814)	70 (5%)	23 (8%)	7 (7%)
	Quetiapine (CSP1027)	101 (6%)	43 (14%)	10 (10%)
	Varenicline (NCT01146613)	102 (7%)	48 (16%)	16 (16%)

*Note:* Demographic information displayed for each cluster among participants that were included in the machine learning model. These data were derived from collating five randomized controlled trials that were designed to assess six different medications for the treatment of alcohol use disorder. Participants with complete drinking data for Days 57–63 but incomplete baseline data were included in the clustering analysis but not in subsequent analyses. Values for age, education and baseline drinks reflect the mean (SD) for each cluster and values for the remaining categorical variables represent frequencies (% of total cluster) within a given cluster.

**TABLE 2:** Participant physiological, biological and clinical characteristics.

		Cluster		
		Mild (*n* = 1531)	Moderate (*n* = 299)	Severe (*n* = 101)
Physiological	Systolic blood pressure	133.3 (17.5)	138.0 (17.7)	136.9 (16.4)
	Diastolic blood pressure	83.6 (10.8)	86.3 (10.8)	84.5 (10.8)
	Pulse rate	73.8 (11.8)	74.5 (12.8)	79.2 (12.2)
	Weight (lbs)	171.5 (48.7)	153.0 (57.8)	179.3 (51.0)
Blood & liver	Creatine levels	0.90 (0.1)	0.87 (0.1)	0.88 (0.1)
	Bilirubin levels	0.56 (0.2)	0.61 (0.3)	0.63 (0.3)
	GGT levels	67.4 (93.4)	77.0 (115.0)	129.9 (297.3)
	ALT levels	37.5 (34.2)	38.7 (28.2)	45.1 (38.5)
	AST levels	33.9 (29.6)	35.1 (22.9)	41.4 (29.9)
Clinical	DrinC score	−0.03 (0.9)	−0.09 (0.9)	0.41 (1.0)
	Depression & anxiety score	−0.03 (0.9)	0 (0.9)	0.35 (1.1)
	CIWA score	−0.04 (0.9)	0.07 (1.0)	0.31 (1.3)

*Note:* Clinical, biological and physiological information displayed by cluster for participants included in the machine learning model. Data were collated from five clinical trials that were designed to assess six different medications for the treatment of alcohol use disorder. Participants with complete drinking data for Days 57–63 but incomplete baseline data were included in the clustering analysis but not in subsequent analyses. Table values represent means (SD) across the three cluster for each assessment. Data for clinical assessments (i.e., DrinC, depression & anxiety, CIWA) reflect data normalized across the entire sample.

### 2.4 Prediction and Characterization of Identified Drinking Patterns

Clinical and physiological data from the first week of treatment for each RCT was used to predict drinking trajectories identified in the DTW clustering using a tree‐based machine learning approach (extreme gradient boosting; XGBoost) [29]. Prior to model building, data were split into a training (70%) and test (30%) such that each set had similar distributions of the three drinking trajectories. In addition, categorical variables were dummy coded and participants with missing feature data were removed (*n*_Full_ = 1931, *n*_Train_ = 1353, *n*_Test_ = 578). Ten‐fold repeated cross‐validation (*n* = 50) was performed to identify optimal hyperparameters for the model using a random search function. Up‐sampling [30] was performed during cross‐validation to avoid prediction bias of the overrepresented pattern (i.e., lowest consumption pattern) by increasing the number of samples in the moderate and severe patterns through duplication. This optimal combination of hyperparameters was then applied to the full training data with up‐sampling to determine the utility of the feature set for predicting the drinking clusters identified. Feature importance was extracted from the final model and was used as a metric for quantifying the effect of a given variable on overall model performance. We tested the generalizability of our model by applying derived model parameters from the tuned model to the test dataset (30% of overall sample) and calculating prediction accuracy.

To assess the sensitivity of our model's performance, we ran an additional gradient boosting model using the same pipeline described above but removed baseline alcohol consumption as a feature. This allowed us to determine whether other clinical outcomes drive prediction in the absence of previous drinking history. To further characterize the clusters, we compared the treatment response (i.e., baseline SDU vs. end‐of‐treatment SDU) of participants within each cluster using a mixed‐effect regression model. This model included the interaction between the phase of the trial, sex and cluster as fixed effects and participant nested within RCT as a random effect to account for differences between the trials. The inclusion of sex in our model allowed us to explore potential sex‐specific effects in treatment response.

## 3 Results

### 3.1 Evaluation of Drinking Patterns

**FIGURE 1:**
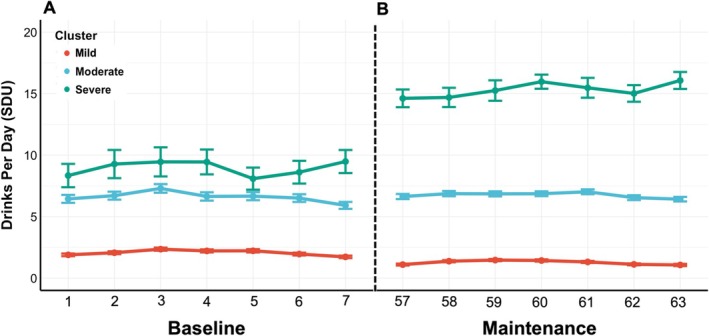
Drinking trajectories by cluster. Line plot displays average standard drinking units (SDU) during (A) Days 1–7 of baseline and (B) Days 57–63 of the treatment for each drinking cluster (i.e., mild, moderate and severe) that was derived from the dynamic time warping (DTW) clustering algorithm.

Results from the three‐cluster solution of drinking rates during Days 57 through 63 of the maintenance phase revealed distinct clusters that were representative of mild, moderate and severe rates of drinking. Tables 1 and 2 display sample characteristics across the identified clusters for participants included in the machine learning model. The COP distance metric for the three‐cluster solution was 0.07 and outperformed a two‐cluster solution (COP = 0.09) where lower COP values indicate a better fit. The cluster representing the lowest rates of drinking had the greatest cluster membership (*n* = 1531) and an average SDU of 1.3 in that 7‐day period, whereas the cluster with moderate drinking rates had a sample size of 299 and an average SDU of 6.7 in that 7‐day period. Finally, the cluster with the highest rates of drinking had the lowest membership (*n* = 101) and an average SDU of 15.3 in that 7‐day period. Figure 1 displays the average SDU for each of the three clusters across the 7 days of baseline (Figure 1A) and treatment (Figure 1B).

### 3.2 Baseline AUD Expression Predicts Treatment Response

After identifying a clustering solution based on participant drinking rates during the end of the maintenance phase of treatment, we sought to determine whether baseline AUD expression, in addition to demographic information, could predict treatment response. Balanced accuracy of the final, tuned model was 82% (F_1_ = 0.63) for the training dataset (*n* = 1353, 70% of entire sample). To assess ‘out‐of‐sample’ generalizability, we used model weights from the tuned model to predict cluster membership in the unseen held‐out test dataset (*n* = 578, 30% of entire sample). Balanced accuracy of the test set was 71% (F_1_ = 0.52) and suggests relatively modest generalizability of our model.

**FIGURE 2:**
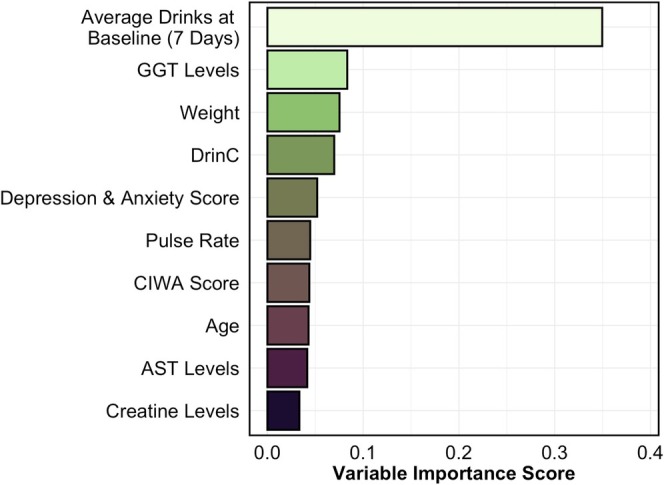
Top 10 variables for cluster prediction. Bar chart displaying top 10 variables that contributed to the classification accuracy of the gradient boosted model for drinking clusters derived from alcohol consumption patterns during Days 57–63 of treatment. Variable importance was extracted from the final model that included optimal hyperparameters.

Tree‐based approaches (e.g., XGBoost) allow for the calculation of feature importance as a way of identifying variables which drove model performance. Figure 2 displays unscaled feature importance scores for the top 10 variables in the fully tuned model. Although average SDU during the first week of the trials had the greatest effect on model performance (Loading = 0.35), other variables among the top 10 represented a constellation of assessments across multiple dimensions that included liver enzyme levels (Loading_GGT_ = 0.08; Loading_AST_ = 0.04), measures of drinking consequences (Loading = 0.07) and physiological measures such as weight (Loading = 0.08) and pulse (Loading = 0.04). Nonetheless, variable importance scores suggest that the primary predictor of alcohol use during treatment was use at the onset of the trials. Of the 13 treatments administered across the trials, none had importance scores over 0.001 except naltrexone (Loading = 0.006) and placebo (Loading = 0.007). Finally, measures including participant sex, having an income between $30 001–$60 000 or not providing an income had loading values of zero. Results from our sensitivity test revealed that removing baseline alcohol consumption as a feature in the model resulted in a 4% decrease in model performance using the training dataset (Balanced Accuracy = 78%; F_1_ = 0.53) and a 10% decrease in model performance on the held‐out test dataset (Balanced Accuracy = 61%; F_1_ = 0.41). The order of importance for variables in this model was generally consistent with those from the primary model with the exception of diastolic and systolic blood pressure now loading as the 9th and 10th important variables, respectively. In addition, liver AST levels were no longer among the top 10 important variables.

**FIGURE 3:**
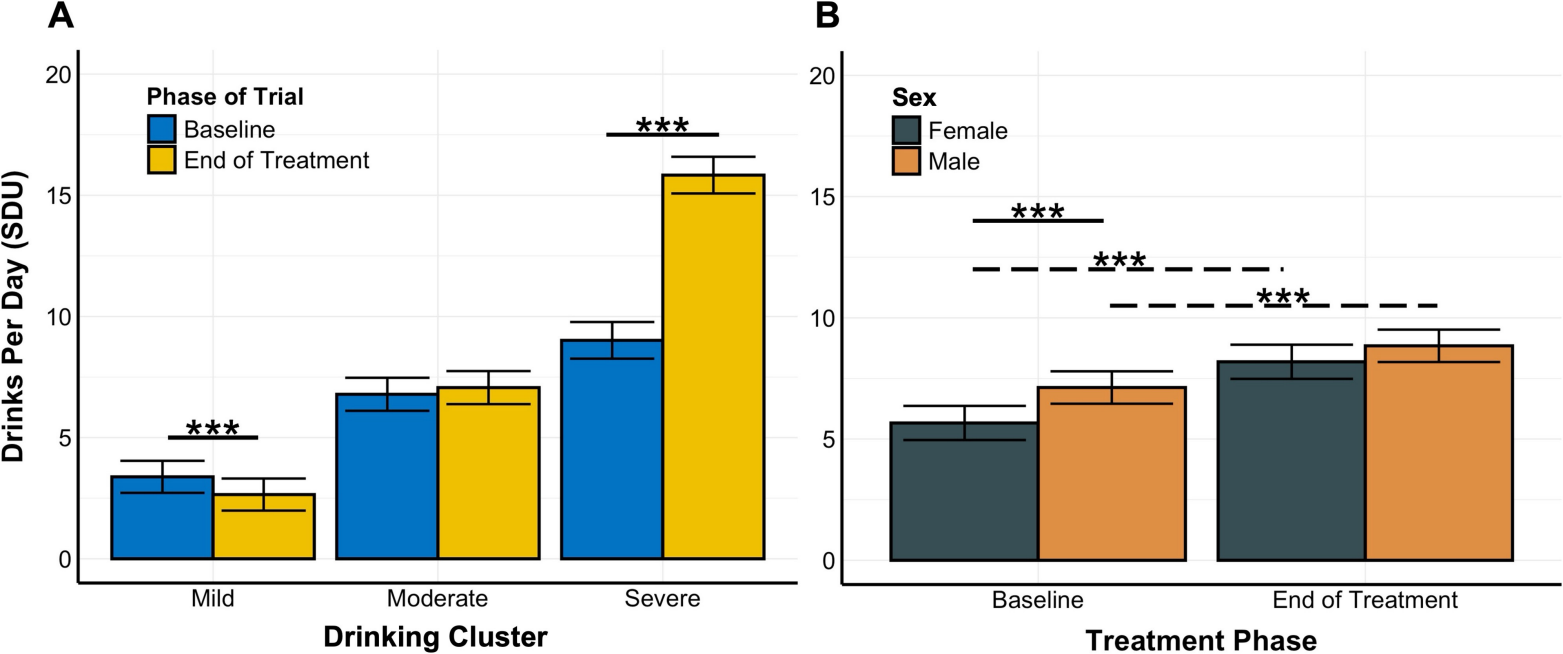
Characterization of drinking clusters and sex‐specific effects. Significant differences in standard drinking units (SDU) were identified from a mixed‐effect model of the interaction between sex, phase of trial and drinking cluster with a random effect for both trial and participant. (A) Bar chart displaying differences in SDU between baseline and the end of treatment across each cluster averaged across sex. (B) Bar chart displaying differences between male and female SDU at baseline and the end of treatment averaged across cluster. Error bars reflect bootstrapped confidence intervals. *Indicates a statistically significant Bonferroni‐corrected comparison from the mixed‐effect models. Dashed lines represent significant within‐sex differences between baseline and the end of treatment. Solid line represents a significant within‐phase difference between males and females.

To provide additional nuance to the relationship between baseline (i.e., primary prediction driver) and end‐of‐treatment drinking (i.e., Days 57–63 that were used to obtain drinking clusters), we first performed a Spearman's correlation between average drinking rates during the first week of each trial and Days 57–63 of treatment. Results from this analysis revealed a correlation of *r* = 0.59 (*p* < 0.001), confirming the strong relationship identified in the machine learning model. Next, we performed a mixed‐effect regression with a nested random effect structure (i.e., participant nested within RCT) to compare differences in treatment response between participants within each cluster (Figure 3). Results from this model revealed that average baseline SDU for the participants in the mild cluster was significantly lower at the end of treatment (*M*_*Difference*_ = −0.731, SE = 0.114, *p* < 0.001) when compared with baseline. However, participants in the most severe cluster had a significant increase in alcohol consumption from baseline to the end of treatment (*M*_*Difference*_ = 6.82, SE = 0.52, *p* < 0.001). No differences were observed for participants in the moderate cluster. Together, these findings suggest that while participants in the mild cluster reduced their drinking from baseline by 22%, those in the severe cluster escalated their use by 75% at the end of treatment.

Finally, due to well‐established differences in drinking rates that are observed between males and females, we hypothesized that participant sex would emerge as a strong driver of prediction in our model. However, feature importance scores for male and female distinctions in our model were zero, indicating no influence of sex on prediction. As an additional exploratory analysis, sex was included as a fixed effect three‐way interaction term in the mixed‐effects regression described above. Results from this analysis revealed that males had significantly higher rates of drinking at baseline (*M*_*Difference*_ = 1.46, SE = 0.287, *p* < 0.001) when compared with females. Additional post hoc comparisons suggest the significant difference at baseline was likely driven by higher rates of consumption for males within the moderate (*M*_*Difference*_ = 1.33, SE = 0.379, *p* < 0.006) and severe clusters (*M*_*Difference*_ = 2.81, SE = 0.755, *p* < 0.003). No significant differences (*M*_*Difference*_ = −0.658, SE = 0.287, *p* = 0.131) were observed between males and females at the end of treatment (Figure 3b). Post hoc comparisons for within‐sex differences in alcohol consumption between phases of the RCTs revealed that both males (*M*_*Difference*_ = 1.72, SE = 0.184, *p* < 0.001) and females (*M*_*Difference*_ = 2.52, SE = 0.355, *p* < 0.001) increased their drinking between baseline and the end of treatment. However, females in the severe group had a greater increase in alcohol consumption from baseline to the end of treatment (*M*_*Difference*_ = 7.58 SDU, SE = 0.948, Cohen's *D* = 0.33) when compared with males (*M*_*Difference*_ = 6.10 SDU, SE = 0.456, Cohen's *D* = 0.36).

## 4 Discussion

Our primary goal was to identify subtypes of AUD treatment response derived from patterns of drinking reported near the end of treatment and predict these subtypes using a constellation of data collected at baseline. Using combined data from five heterogeneous clinical trials designed to evaluate the efficacy of different medications for AUD, we identified three distinct clusters of alcohol consumption derived from drinking reported during a final week of treatment (i.e., Days 57–63 of treatment). Cluster membership was representative of AUD prevalence rates in the general population [31] such that the most severe drinking cluster had the fewest individuals (6%) whereas the cluster with the lowest rates of drinking contained a majority of the participants. Our cross‐validated machine learning approach achieved modest predictive accuracy of these clusters using core demographics and baseline clinical assessments as features. Follow‐up analyses revealed that model performance was driven by changes in alcohol consumption between baseline and the end of treatment for the mild and severe clusters.

Classification accuracy (i.e., balanced accuracy and F_1_) of our tree‐based machine learning model was primarily driven by drinking rates at the start of the trial (i.e., prior to receiving a full dose of medication) followed by core alcohol‐related clinical assessments and liver functioning. Baseline alcohol consumption emerging as the most important predictor of drinking patterns at the end treatment was unsurprising given prior findings on predictors of AUD treatment success [21, 32, 33]. Removing baseline drinking as a feature in the model—as a form of sensitivity analysis—resulted in a 10% drop in test set classification accuracy. However, the performance of this model without baseline consumption was driven by similar physiological and clinical features (i.e., weight, GGT, AST and DrinC) as the model that included baseline drinking. Although this finding is consistent with prior machine learning studies [5, 20, 21], the effect of baseline drinking may have been more pronounced in our generalization test due to the lack of treatment effects that were observed for most of the medications tested in each trial. For example, of the seven medications (including the combination of naltrexone and acamprosate) evaluated across the five RCTs, only varenicline [19] and naltrexone [15] were shown to reduce problematic drinking.

This lack of treatment effect may have also been exacerbated by low rates of drinking that were reported during the trials. Indeed, our clustering analysis revealed that most participants had mild rates of drinking during the entire length of the trials. Follow‐up comparisons of baseline and end‐of‐treatment SDU suggest that participants in the mild drinking cluster reduced their drinking by 22% at the end of treatment, and those in the most severe group increased their drinking by 75%. Taken together, this might suggest that the individuals with more mild forms of AUD improved over the course of treatment irrespective of the active medication conditions or placebo they received.

Prior work has shown that males report higher rates of alcohol consumption than females and are more likely to be diagnosed with AUD. However, recent data suggest that males and females report consuming similar amounts of alcohol while enrolled in clinical trials for treatment of AUD [34]. Our results, derived from data collated across five separate trials, support these recent findings. For example, within the clusters we identified, females had significantly lower rates of drinking at baseline but reported comparable rates to males by the end of treatment (i.e., Days 57–63). This finding was further supported by the near‐zero effect of sex on predicting cluster membership in our machine learning model. Although the relative quantity of alcohol consumed was the same between sexes by the end of treatment, females are more likely to have a higher metabolic response and report greater subjective effects after consuming the same amount of alcohol because of sex‐specific differences in alcohol pharmacokinetics [35]. Thus, females across these trials may have had worse AUD‐related outcomes compared with males while endorsing the same level of consumption. Furthermore, post hoc analyses of within‐sex differences between the drinking rates at baseline and the end of treatment suggest that, in addition to worse AUD‐related outcomes, females with severe AUD had greater escalations in drinking when compared with males with severe AUD. This suggests that the lack of sex‐specific differences in drinking at the end of treatment may be due to escalation in use among females with severe AUD. Outcomes such as these highlight the need to prospectively consider sex differences when designing medication trials for AUD and to analyse and present data by sex [36].

This study has several limitations that must be addressed. First, although combining multiple heterogenous RCTs is a strength of this study, a majority of the sample were participants from the COMBINE study (NCT00006206), which may have biased cluster assignment and feature importance from the machine learning model. Furthermore, individuals with missing data were removed, which may have biased our sample towards a more treatment compliant sample with less severe forms of AUD. Thus, these clusters may not necessarily generalize to other AUD populations, and future work should consider clustering methods that include cross and external validation [37, 38, 39]. Nonetheless, we achieved relatively modest prediction accuracy of our clusters with an unseen, held‐out test dataset that was driven by metrics of alcohol use severity (i.e., baseline use and drinking consequences). Second, because there was a lack of harmonization between some aspects of the RCTs, composite scores were created for the anxiety and depression measure, and factor levels were combined for several demographic variables such as income and marital status. This may have diminished the effect of these features on overall model performance. In addition, due to differences across the RCTs in the number of times certain assessments were performed, and when they were administered, the present analysis was limited in the scope of questions that could be answered. Given well‐established associations between psychopathology and AUD severity, the availability of additional clinical assessments may have increased the performance of the machine learning model. Finally, across the clinical trials, participants were required to endorse at least moderate AUD to be eligible for enrolment. However, participants were recruited across each trial that reported not having consumed alcohol during any treatment phase. In the present analysis, participants who did not report drinking alcohol at the end of treatment (i.e., the days selected for the present analysis) were grouped within the mild cluster by the timeseries‐based clustering approach. The inclusion of individuals that endorsed mild forms of drinking may have also been problematic given the likelihood of ‘floor’ effects in the primary outcomes (i.e., binge drinking and abstinence) that may preclude identification of medication effects and associations with AUD‐related phenotypes.

In conclusion, our work demonstrates the feasibility of combining data from multiple clinical trials to identify predictors of treatment response from a highly heterogenous sample of patients. Despite differences in the medications administered, treatment length and patient characteristics, we show that it is possible to derive unique, clinically relevant alcohol use trajectories by leveraging the temporal dynamics of drinking behaviour during clinical trials. These use trajectories (i.e., clusters) we identified map to AUD subpopulations that include individuals with low rates of drinking who tend to reduce consumption during treatment, individuals who do not change their consumption pattern during treatment, and individuals who have high rates of drinking at the start of treatment and continue to escalate their use. Furthermore, the predictive performance of our machine learning model on a held‐out sample was comparable with model performance during training and indicates high generalizability of our findings. This highlights further both the feasibility of combining heterogenous sets of data to better elucidate AUD‐related outcomes and the utility of using the temporal dynamics of drinking behaviour to identify more generalizable and thus more reproducible findings. Given that these models often require thousands of samples, future work should consider leveraging data harmonization methods to better understand AUD‐related phenotypes and their treatment.

## Author Contributions

**Robert J. Kohler:** conceptualization (lead), data curation (lead), formal analysis (lead), writing – original draft (lead), writing – review and editing (lead). **Yasmin Zakiniaeiz:** conceptualization (equal), writing – review and editing (equal). **Terril L. Verplaetse:** conceptualization (equal), writing – review and editing (equal). **C. Leonardo Jimenez Chavez:** writing – review and editing (equal). **MacKenzie R. Peltier:** conceptualization (equal), writing – review and editing (equal). **Hang Zhou:** conceptualization (equal), writing – review and editing (equal). **Sherry A. McKee:** conceptualization (equal), funding acquisition (equal), writing – review and editing. **Walter Roberts:** conceptualization (equal), data curation (equal), writing – original draft (equal), writing – review and editing.

## Conflicts of Interest

The authors declare no conflicts of interest.

## Supporting information


**Table S1** Demographic Characteristics by Randomized Controlled Trial. Table displays demographic characteristics across each randomized controlled trial after removing participants with missing features. Data shown here reflects that which was included in the machine learning model and subsequent follow‐up analyses. Levels for categorical variables including employment, marital status, race and ethnicity were combined for some RCTs due to differences in the number of response categories for a given variable.
**Table S2.** Clinical and Biological Descriptive Statistics by Randomized Controlled Trial. Table displays baseline biological and clinical characteristics across each randomized controlled trial after removing participants with missing features. Data shown here reflects that which was included in the machine learning model and subsequent follow‐up analyses. Data for Drinking Consequences (DrinC), Depression & Anxiety (Composite Scores) and CIWA were normalized prior to any analysis due to differences in scale across the randomized controlled trials for these variables. Table values represent Means (SD).

## Data Availability

The data that support the findings of this study are available from NIAAA. Restrictions apply to the availability of these data, which were used under license for this study. Data are available from https://nda.nih.gov/niaaa with the permission of NIAAA.
