# Supplementary material for: Empirical Derivation and Prediction of Treatment Trajectories in Harmonized AUD Clinical Trial Datasets
Source: Addict Biol. 2025 Jul 23;30(7):e70069. doi: 10.1111/adb.70069 (PMC12285684; doi:10.1111/adb.70069)
Supplement: Supplementary file 1 — Table S1 Demographic Characteristics by Randomized Controlled Trial. Table displays demographic characteristics across each randomized controlled trial after removing participants with missing features. Data shown here reflects that which was included in the machine learning model and subsequent follow‐up analyses. Levels for categorical variables including employment, marital status, race and ethnicity were combined for some RCTs due to differences in the number of response categories for a given variable. Table S2. Clinical and Biological Descriptive Statistics by Randomized Controlled Trial. Table displays baseline biological and clinical characteristics across each randomized controlled trial after removing participants with missing features. Data shown here reflects that which was included in the machine learning model and subsequent follow‐up analyses. Data for Drinking Consequences (DrinC), Depression & Anxiety (Composite Scores) and CIWA were normalized prior to any analysis due to differences in scale across the randomized controlled trials for these variables. Table values represent Means (SD). [file ADB-30-e70069-s001.docx]

**SUPPLEMENTAL MATERIAL**

**1. CHARACTERISTICS OF RANDOMIZED CONTROLLED TRIALS**

***1.1 COMBINE (NCT00006206)***

NCT00006206 was a Phase 3 multi-site trial sponsored by the NIAAA with the goal of evaluating acamprosate and naltrexone for the treatment of AUD in 1383 individuals who met criteria for alcohol dependence. In addition, eligible participants were also required to be seeking treatment and recently abstinent. The trial lasted a total of 16 weeks with primary data collection occuring at baseline, week 8 and week 16. Participants could be randomized into one of the following conditions, naltrexone (n=154), acamprosate (n=152), naltrexone + acamprosate (n=148), combined behavioral therapy (CBI) alone (n=157) and in a subset of individuals receiving naltrexone (n=155), acamprosate (n=151), and the combination (n=157). A subset of participants was also randomized to a placebo group or placebo in combination with CBI. All participants except for those in the CBI only group received 9 sessions of a Medical Management intervention at weeks 0, 1, 2, 4, 6, 8, 10, 12 and 16. Maintenance phase of treatment began on week 2 after 7 days of dose escalation for both all medication combinations. For additional information see Anton et al., 2006.

***1.2 Gabapentin (NCT01613014)***

NCT01613014 was a Phase 2 multi-site trial affiliated with NIAAA’s Clinical Investigation Group (NCIG) designed to evaluate the efficacy of Horizant (Gabapentin XR; N=338) among individuals with at least moderate AUD over a 26-week period. Participants were randomized to receive Gabapentin XR (n=170) or placebo (n=168) following three consecutive days of abstinence. Dose escalation took place during the first week of treatment with maintenance phase beginning on week two and ending on week 25. All enrolled participants were required to view a computerized bibliotherapy platform, “Take Control”, at each visit. For additional information see Falk et al., 2019

***1.3 Levetiracetam (NCT00970814)***

NCT00970814 was a Phase 2 muti-site trial affiliated with NIAAA’s Clinical Investigation Group (NCIG) designed to evaluate the efficacy of Levetiracetam (N=130) in heavy drinkers over 16 weeks. Participants were randomized to receive an extended-release formulation of Levetiracetam (n=64) or placebo (n=66). Dose escalation took place during the first four weeks of treatment with maintenance phase beginning on week five and ending at the start of week 15. In clinic assessments occurred at screening, baseline, and during weeks 2, 3, 4, 6, 8, 10, 12, 14, 15 and 17. All participants also received 11 sessions of the Brief Behavioral Compliance Enhancement Treatment intervention. For additional information see Fertig et al., 2012.

***1.4 Quetiapine (CSP1027)***

CSP1027 was a Phase 2 multi-site trial affiliated with NIAAA’s Clinical Investigation Group (NCIG) designed to evaluate the efficacy of Quetiapine (N=218) in heavy drinkers. Participants were not required to be abstinent for any period of time prior to baseline. Eligible individuals were randomized to receive either Quetiapine (n=105) or placebo (n=113) over a 13-week period. All enrolled participants were also required to complete a Medical Management intervention at each in person visit (n=9 total visits). Maintenance phase of treatment began on week three of the trial after two weeks of dose escalation. All clinical and biological assessment data were administered at screening, baseline, and weeks 2, 3, 4, 6, 8, 10, 12 and 13. For additional information see Litten et al., 2012

***1.5 Varenicline (NCT01146613)***

NCT01146613 was a Phase 2 multi-site trial affiliated with NIAAA’s Clinical Investigation Group (NCIG) designed to evaluate the efficacy of Varenicline (N= 200) among individuals meeting criteria for alcohol dependence. Participants were randomized to receive Varenicline (n=99) or placebo (n=101) over 13 weeks. In-person clinical data was collected during screening, baseline and weeks 2, 4, 6, 10, and 14. In addition to receiving Varenicline or placebo, Maintenance phase began on week two following a week of dose escalation and ended on week 13. All enrolled participants were required to view a computerized bibliotherapy platform, “Take Control”, which consisted of six separate treatment modules. A single module was completed at each of the 6 in person visits. For additional information see Litten et al., 2013

**2. DATA HARMONIZATION**

***2.1 Demographic Variables***

While demographic information that was collected across the RCTs was generally consistent, several RCTs had more response options for some demographic items. Thus, harmonization for each demographic variable was based on the RCT with the fewest available response options for a given variable. In the case of yearly income, some RCTs allowed for participants to indicate an annual income of $30,001-$60,000 whereas another may have included options for $30,001-$45,000 and $45,001-$60,000. Thus, we chose four stratifications for annual income that included “<$30,000”, “$30,001-$60,00”, “>$60,000” and “Not Provided”. For marital status, some of the RCTs included an option for those who were widowed, living with their partner, or separated. To account for increased stratification, we chose stratifications of “Married” and “Not Married”/“Other”. For employment status, participants that indicated any full- or part-time employment were considered “Working” as opposed to “Unemployed”/“Other”, and cases for options such as student or military were coded as “Other”. Finally, race and ethnicity response options were highly heterogenous and thus coded as either “white” or “non-white” for race stratification and “Hispanic“ or “Non-Hispanic” for stratification of ethnicity.

***2.2 Biological and Clinical Variables***

In order to include a measure of psychopathology in our analyses, we were required to create a composite score using data from surveys that include items related to psychopathology. Across each of the five 5 RCTs, we identified survey items pertaining to measures of anxiety and depression that could be used to create unique composite scores for each RCT. This included survey items from the Short Form-12 assessment for NCT01146613, the Hamilton Anxiety Scale and Montgomery Asberg Depression Rating Scale for NCT00970814 and CSP1027, Beck Depression and Anxiety Indices for NCT01613014 and the Brief Symptom Inventory for NCT00006206. Prior to collating data across all the RCTs, survey items were summed for each trial separately and then scaled such that the new composite scores were centered at zero with a standard deviation of one.

**Supplemental Table 1.**

|  | **COMBINE (NCT00006206) (N=1216)** | **Gabapentin (NCT01613014) (N=295)** | **Levetiracetam (NCT00970814) (N=100)** | **Quetiapine (CSP1027) (N=154)** | **Varenicline (NCT01146613) (N=166)** |
| --- | --- | --- | --- | --- | --- |
| **Age** |  |  |  |  |  |
| Mean (SD) | 44.6 (10.3) | 50.8 (10.4) | 45.3 (11.8) | 45.4 (10.3) | 45.5 (11.7) |
| **Gender** |  |  |  |  |  |
| Female | 378 (31.1%) | 98 (33.2%) | 24 (24.0%) | 33 (21.4%) | 48 (28.9%) |
| Male | 838 (68.9%) | 197 (66.8%) | 76 (76.0%) | 121 (78.6%) | 118 (71.1%) |
| **Education (Years)** |  |  |  |  |  |
| Mean (SD) | 14.6 (2.70) | 15.3 (2.73) | 13.9 (2.47) | 13.8 (2.74) | 14.5 (2.96) |
| **Race** |  |  |  |  |  |
| Non-White | 284 (23.4%) | 78 (26.4%) | 38 (38.0%) | 24 (15.6%) | 62 (37.3%) |
| White | 932 (76.6%) | 217 (73.6%) | 62 (62.0%) | 130 (84.4%) | 104 (62.7%) |
| **Ethnicity** |  |  |  |  |  |
| Hispanic | 135 (11.1%) | 25 (8.5%) | 1 (1.0%) | 4 (2.6%) | 3 (1.8%) |
| Non-Hispanic | 1081 (88.9%) | 270 (91.5%) | 99 (99.0%) | 150 (97.4%) | 163 (98.2%) |
| **Cluster Membership** |  |  |  |  |  |
| Mild | 1071 (88.1%) | 187 (63.4%) | 70 (70.0%) | 101 (65.6%) | 102 (61.4%) |
| Moderate | 87 (7.2%) | 98 (33.2%) | 23 (23.0%) | 43 (27.9%) | 48 (28.9%) |
| Severe | 58 (4.8%) | 10 (3.4%) | 7 (7.0%) | 10 (6.5%) | 16 (9.6%) |
| **Marriage Status** |  |  |  |  |  |
| Married | 513 (42.2%) | 145 (49.2%) | 36 (36.0%) | 65 (42.2%) | 65 (39.2%) |
| Not married | 703 (57.8%) | 150 (50.8%) | 64 (64.0%) | 89 (57.8%) | 101 (60.8%) |
| **Employment** |  |  |  |  |  |
| Unemployed/Other | 482 (39.6%) | 73 (24.7%) | 32 (32.0%) | 47 (30.5%) | 50 (30.1%) |
| Working | 734 (60.4%) | 222 (75.3%) | 68 (68.0%) | 107 (69.5%) | 116 (69.9%) |
| **Income** |  |  |  |  |  |
| <$30,000 | 319 (26.2%) | 69 (23.4%) | 43 (43.0%) | 49 (31.8%) | 55 (33.1%) |
| $30,001-$60,000 | 359 (29.5%) | 46 (15.6%) | 17 (17.0%) | 49 (31.8%) | 33 (19.9%) |
| >$60,000 | 526 (43.3%) | 165 (55.9%) | 40 (40.0%) | 55 (35.7%) | 76 (45.8%) |
| Not Provided | 12 (1.0%) | 15 (5.1%) | 0 (0%) | 1 (0.6%) | 2 (1.2%) |
| **Baseline SDU**  **(Days 1 - 7)** |  |  |  |  |  |
| Mean (SD) | 1.33 (2.66) | 4.37 (3.20) | 8.22 (4.79) | 5.84 (5.76) | 8.42 (6.59) |
| **Maintenance SDU (Days 57 - 63)** |  |  |  |  |  |
| Mean (SD) | 2.13 (4.21) | 3.88 (2.96) | 4.06 (4.16) | 3.68 (3.84) | 4.79 (4.82) |

**Supplemental Table 1. Demographic Characteristics by Randomized Controlled Trial.** Table displays demographic characteristics across each randomized controlled trial after removing participants with missing features. Data shown here reflects that which was included in the machine learning model and subsequent follow-up analyses. Levels for categorical variables including employment, marital status, race, and ethnicity were combined for some RCTs due to differences in the number of response categories for a given variable.

**Supplemental Table 2.**

|  | **COMBINE (NCT00006206) (N=1216)** | **Gabapentin (NCT01613014) (N=295)** | **Levetiracetam (NCT00970814) (N=100)** | **Quetiapine (CSP1027) (N=154)** | **Varenicline (NCT01146613) (N=166)** |
| --- | --- | --- | --- | --- | --- |
| **Systolic Blood Pressure** | 134 (18.2) | 137 (16.5) | 137 (14.9) | 133 (17.7) | 133 (16.0) |
| **Diastolic Blood Pressure** | 83.5 (11.3) | 85.9 (9.74) | 86.1 (9.45) | 84.3 (11.8) | 83.9 (9.80) |
| **Pulse Rate** | 75.3 (12.1) | 70.5 (10.9) | 73.5 (12.3) | 77.3 (12.5) | 71.2 (11.3) |
| **Weight (lbs)** | 180 (38.8) | 88.8 (20.3) | 195 (38.9) | 188 (45.1) | 197 (41.2) |
| **Creatine** | 0.90 (0.17) | 0.88 (0.18) | 0.91 (0.17) | 0.89 (0.16) | 0.88 (0.15) |
| **Total Bilirubin** | 0.56 (0.25) | 0.58 (0.27) | 0.59 (0.35) | 0.58 (0.27) | 0.58 (0.25) |
| **Gamma-Glutamyl Transferase** | 75.3 (128) | 51.3 (51.1) | 69.8 (74.2) | 92.6 (145) | 69.3 (102) |
| **Alanine Aminotransferase** | 40.3 (37.2) | 31.7 (23.6) | 35.4 (26.3) | 37.8 (26.3) | 35.3 (29.9) |
| **Aspartate Aminotransferase** | 36.2 (33.2) | 29.8 (17.5) | 32.7 (16.4) | 33.5 (19.5) | 32.6 (20.3) |
| **Drinking Consequences (DrinC)** | -0.01 (1.00) | -0.06 (0.94) | -0.09 (0.89) | 0.02 (1.02) | 0.03 (0.98) |
| **Depression & Anxiety** | -0.007 (0.99) | -0.041 (0.97) | 0.027 (0.97) | -0.028 (1.03) | 0.031 (1.01) |
| **Withdrawal (CIWA)** | 0.003 (1.01) | -0.003 (1.03) | -0.032 (0.94) | -0.0440 (0.97) | -0.006 (0.97) |

**Supplemental Table 2. Clinical and Biological Descriptive Statistics by Randomized Controlled Trial.** Table displays baseline biological and clinical characteristics across each randomized controlled trial after removing participants with missing features. Data shown here reflects that which was included in the machine learning model and subsequent follow-up analyses. Data for Drinking Consequences (DrinC), Depression & Anxiety (Composite Scores), and CIWA were normalized prior to any analysis due to differences in scale across the randomized controlled trials for these variables. Table values represent Means (SD).
